# Participation and Experiences in Extracurricular Activities for Children With Developmental Language Disorder and Their Peers

**DOI:** 10.1111/1460-6984.70134

**Published:** 2025-10-05

**Authors:** Callyn Farrell, Virginia Slaughter, Aisling Mulvihill

**Affiliations:** ^1^ School of Psychology The University of Queensland Brisbane Australia; ^2^ Queensland Brain Institute The University of Queensland Brisbane Australia

**Keywords:** barriers, developmental language disorder, extracurricular activities, facilitators, leisure activities, participation

## Abstract

**Objectives:**

Participation in organised extracurricular social activities (OESAs) can provide wide‐ranging positive developmental benefits for children. This study investigated whether participation and experiences differ for children aged 4‐ to 12‐years with a developmental language disorder (DLD) compared to their typical language developing (TLD) peers.

**Methods:**

Parents of children with DLD (n = 18) and those of TLD peers (n = 21) reflected on their child's participation and experiences in OESAs.

**Results:**

Results demonstrated that parents of children with DLD reported engagement in a similar number of OESAs and for a similar length of time on a weekly basis compared to parents of TLD children. Additionally, when evaluating factors that facilitated positive participation experiences— such as their child's abilities and behaviour, features of the OESA, and the social environment —parents of children with DLD provided ratings mostly comparable to those of TLD parents. However, when children disengaged from an OESA, ability‐related factors, such as communication, motor, and social skills, were more likely to be reported to influence the participation experience for children with DLD.

**Conclusions:**

These findings underscore the importance of fostering accessible and positive OESA experiences to support meaningful participation and access to the developmental benefits of OESAs for children with DLD.

**WHAT THIS PAPER ADDS:**

*What is already known on this subject*

- Children with developmental disabilities often experience reduced participation in organised extracurricular social activities (OESAs), limiting their access to important developmental opportunities. For children with developmental language disorder (DLD), research has focused primarily on academic challenges, with limited understanding of their participation and experiences in non‐academic, socially orientated activities. Previous studies have generally assumed participation barriers dominate the experiences of children with disabilities, potentially overlooking strengths, facilitators, and positive developmental contexts like OESAs.

*What this study adds to existing knowledge*

- This study provides the first comprehensive comparison of OESA participation and experiences between children with DLD and their typically developing peers. Findings suggest that children with DLD participate at similar rates, intensities, and breadths and generally have positive experiences. Ability‐related factors such as communication and motor skills influenced disengagement but did not preclude participation. By applying a biopsychosocial framework, this study moves beyond a deficit‐based view and highlights the potential for inclusive OESA environments to support social development for children with DLD.

*What are the potential or actual clinical implications of this study?*

- OESAs offer developmentally rich contexts that may support social engagement and psychosocial well‐being for children with DLD. Clinicians and educators should encourage participation in OESAs while being mindful of subtle ability‐related challenges that may affect sustained engagement. Targeted support strategies—such as facilitating beginner‐friendly entry points and fostering inclusive peer environments — could optimise participation. These findings highlight the importance of advocating for accessible, socially supportive community programmes as part of holistic developmental support plans for children with DLD.

## 1 Introduction

Organised extracurricular social activities (OESAs) are leisure activities that take place outside formal schooling and involve structured social interaction with peers, coaches, or facilitators. OESAs provide a community‐based biopsychosocial developmental opportunity for all children, including children with developmental difficulties (Bohnert et al. 2019; McCabe et al. 2016). For children with developmental language disorder (DLD), who experience persistent expressive and/or receptive language difficulty, participation in OESAs may provide a context to develop prosocial skills and healthy peer relationships (Oberle et al. 2019; Wright et al. 2010) outside of linguistically demanding academic contexts (Ziegenfusz et al. 2022). Although children with a disability are generally found to show reduced participation in OESAs (Farrell et al. 2024), little is known about the experience of children with DLD specifically (Kentiba 2015; Wachsmuth et al. 2023). This study aimed to (1) investigate whether children with DLD participate in OESAs similar to their typically language developing (TLD) peers and (2) explore the experience of OESA participation for children with DLD in regard to their abilities and behaviour, the OESA programme and environment, and their social experiences in an OESA.

### 1.1 Defining OESAs

OESAs encompass various options, including sports, dance, and art classes. Importantly, the term OESA broadens the scope of children's leisure activities examined in previous research by emphasising those that involve social engagement. Various terms—extracurricular activities, leisure activities, organised programmes, out‐of‐school recreation, and after‐school activities—have been used interchangeably throughout the literature. However, OESAs are distinct in that they are structured, formally organised, and supervised by an adult, coach, or facilitator. Participation is voluntary, occurs regularly over an extended period (e.g., a season), and occurs outside formal school hours. A defining feature of OESAs is their inherently social nature, offering children opportunities to interact, connect, and learn alongside peers, friends, or adults. These activities span various domains, including sports, academics, the arts, social groups, religious programmes, and community‐based initiatives, and the definitions and distinctions outlined here draw heavily on the framework developed by Farrell and colleagues (2024).

### 1.2 Benefits of OESA Participation

Participation in OESAs provides children with opportunities to develop psychosocial and developmental skills, such as a sense of connection, self‐efficacy, and autonomy, which in turn contribute to improved social, emotional, behavioural, academic, and psychological outcomes for all children, regardless of ability (Bohnert et al. 2019; Farrell et al. 2024; Hynes and Block 2022; May et al. 2021; McCabe et al. 2016; Peck et al. 2008). For school‐aged children, OESA participation has been shown to decrease loneliness and increase peer connectedness, belonging, well‐being, social functioning, and emotional adjustment (Oberle et al. 2019). Therefore, OESAs offer a broad range of biopsychosocial benefits that may extend beyond the activities themselves and positively influence children's daily lives.

### 1.3 OESA Participation for Children With Developmental Disabilities

Children with developmental disabilities can also derive benefits similar to their peers. Physical activity‐focused OESAs for children with developmental disabilities may accrue positive developmental benefits (e.g., motor, social and communication skills) as a result of their participation (see Huang et al. 2020 for a review and a meta‐analysis). Additionally, children's participation in OESAs focused on creative movement (e.g., dance) positively benefited their cognitive and social skill development and general psychological functioning (see May et al. 2021 for review and meta‐analysis). Concerningly, while the benefits of OESA participation for children are seemingly numerous, children with developmental disabilities and their parents generally report reduced participation in these activities (Engel‐Yeger et al. 2009; Farrell et al. 2024; King et al. 2010; Law et al. 2011). Such findings raise concern, as this may limit children's (including those diagnosed with DLD) opportunities to derive wide‐ranging biopsychosocial benefits of participation in OESAs throughout childhood.

### 1.4 DLD

DLD is a neurodevelopmental condition characterised by persistent difficulties understanding and/or using language that impacts an individual's everyday life. Further, these difficulties are not better explained by coexisting conditions related to other neurodevelopmental diagnoses (i.e., autism; Bishop et al. 2017; Norbury et al. 2016). Although prevalence studies indicate that approximately 7% of children meet the diagnostic criteria for DLD (Calder et al. 2022; Norbury et al. 2016), only 20% of Australians are aware of this diagnostic label (Kim et al. 2022). Notably, children with DLD are at a higher risk of poorer mental health outcomes (Yew and O'Kearney 2013), lower self‐esteem (Jerome et al. 2002), impaired social cognitive development (i.e., theory of mind; Nilsson and de Lopez 2016), impaired social interactions (Wieczorek et al. 2024) and poorer academic outcomes (Dockrell et al. 2011), when compared to their TLD peers. Importantly, for research regarding OESA participation, children with DLD may also experience difficulties or exhibit deficits in fine and gross motor and coordination skills (Varuzza et al. 2022).

Despite these difficulties, emerging research indicates that prosocial behaviours may act as a protective factor for children with DLD by buffering against social, emotional, and behavioural difficulties (Conti‐Ramsden et al. 2018; Mok et al. 2014; Pickles et al. 2016). Toseeb and St Clair (2020) found that higher levels of prosociality in middle childhood were associated with fewer concurrent social and emotional difficulties in children at risk of DLD. Furthermore, positive early language and communication environments may foster competent social play and higher prosociality in childhood, decreasing externalising challenges throughout development (Toseeb et al. 2020). These findings underscore the potential of prosocial behaviours to mitigate behavioural challenges commonly associated with DLD. Participation in OESAs, which promote and provide opportunities to practise prosocial interactions, may offer valuable experiences for children with DLD.

### 1.5 OESA Participation for Children With DLD

OESAs may provide opportunities for children with DLD to develop social, emotional, and cognitive skills in settings that may impose a lower linguistic load than formal academic contexts, such as school. For example, Ziegenfusz et al. (2022) found that students with DLD experienced difficulties across several academic domains, particularly in literacy‐related tasks, highlighting the linguistic demands of school‐based learning. In contrast, McGregor et al. (2023) reported that children with DLD showed relative strengths in areas such as play, coping, gross motor function and prosocial qualities, domains often less dependent on language. Hence, school settings often require children to navigate complex linguistic demands in structured learning environments, whereas OESAs may provide a more accessible context for children with DLD to engage in meaningful social interactions, develop self‐efficacy, and cultivate prosocial behaviours (McGregor et al. 2023; Ziegenfusz et al. 2022). Given that children with DLD experience persistent difficulties in language comprehension, expression, and social communication (Bishop et al. 2017), structured non‐academic activities may offer a more inclusive and beneficial avenue for participation and skill development (Bohnert et al. 2019; McGregor et al. 2023; Peck et al. 2008).

Despite the potential benefits of OESA participation, research on children with DLD in these contexts is almost non‐existent. Existing studies on extracurricular participation among children with disabilities often group DLD within broad neurodevelopmental categories (e.g., Kentiba 2015; Wachsmuth et al. 2023), obscuring the distinct experiences of this population. Moreover, research in this area has predominantly taken a deficit‐based perspective, emphasising barriers rather than facilitators of participation (Shields et al. 2012), reinforcing assumptions that OESA involvement may be inherently challenging for children with DLD, rather than recognising that such activities may be both challenging and beneficial, offering important developmental opportunities when appropriate. Previous research approaches potentially overlook the unique facilitators and barriers shaping participation for children with DLD and make it difficult to determine whether and how OESAs may serve as a protective and enriching context.

### 1.6 Models of Disability

While contemporary research increasingly embraces multidimensional frameworks, such as the ICF‐CY (e.g., Farrell et al. 2024; World Health Organization 2007), historically, disability experiences have been investigated using medical and social models of disability. The medical model conceptualises disability as an individual condition leading to functional impairments, whereas the social model views disability as a societal construct arising from differential impairments (Haegele and Hodge 2016). While each model offers valuable insights, both present a limited perspective on the complex and individualised nature of disability. The biopsychosocial model integrates these approaches by recognising the interplay between an individual's impairments and their social context (Castro et al. 2011).

Grounded in a biopsychosocial model, the ICF‐CY provides a multidimensional framework for understanding functioning, emphasising participation in activities such as OESAs for children with DLD (World Health Organization 2007). The ICF‐CY consists of two overarching domains, functioning/disability and context (Adolfsson et al. 2011), which are further divided into four components: (1) body, which includes psychological, physical, and sensory functioning, along with body structures; (2) activities and participation, which covers engagement in daily activities; (3) environmental factors, which include social, attitudinal, and physical aspects of a child's environment; and (4) personal factors, which include demographic and individual‐level characteristics (Adolfsson et al. 2011; Ibragimova et al. 2009).

**FIGURE 1:**
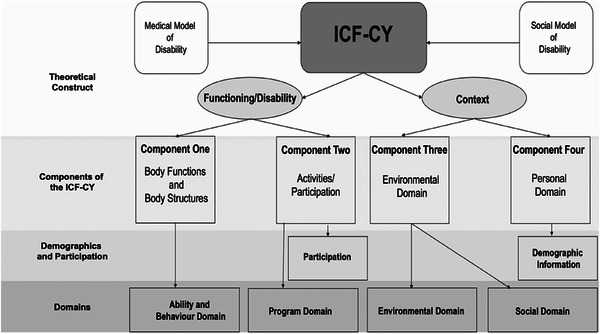
A structural representation of the ICF‐CY theoretical construct, its components, and how they inform the measure used in the present study (Farrell et al. 2024).

The ICF‐CY framework enables a holistic examination of how these components shape a child's participation and experiences in daily life. This study applies the ICF‐CY to investigate OESA participation across all four components, informing the questions posed to parents. Further, here, participation was assessed at three levels: fully engaged, referring to regular participation in OESAs; partially engaged, indicating past participation with later withdrawal; and considered engagement, referring to OESAs that were contemplated but not pursued. Additionally, experiences were assessed considering barriers and facilitators across four domains (ability and behaviour, social, programme, and environmental; see Figure 1). Together, this approach provides a comprehensive understanding of factors influencing OESA participation among children diagnosed with DLD and their TLD peers.

### 1.7 The Present Study

To our knowledge, this is the first study to comprehensively investigate OESA participation and experiences, comparing children (aged 5 to 12 years old) with DLD and their TLD peers in an Australian context. Given the limited representation of DLD children in prior OESA research, this study aims to provide a more nuanced understanding of how children with DLD participate in OESAs. The present study used an established OESA participation and experience online self‐report measure completed by a child's parent (see Farrell et al. 2024) to examine several exploratory research questions. Firstly, which OESA categories—Religious, Academic, the Arts, Sports, and Community/Service (Bohnert et al. 2019)—are most reported on at each level of participation, and does this differ between children with DLD and their TLD peers? Secondly, how do OESA participation intensity, breadth, and number of activities compare between these groups? Lastly, what are the barriers and facilitators experienced at each of the three levels of participation (fully engaged, partially engaged, and considered engagement), and does this differ between children with DLD and their TLD peers?

This study aims to move beyond a deficit‐based lens that assumes participation challenges are inevitable for children with DLD. Instead, we sought to identify if any factors support participation and facilitate positive experiences, helping challenge assumptions about exclusion and contribute to developing more inclusive OESA environments. Understanding what enables and restricts participation is crucial for developing evidence‐based interventions, inclusive programming, and policy recommendations that promote equitable access to OESAs for children with language impairments.

## 2 Methods

### 2.1 Participants

The final dataset represents 39 Australian parents (total sample: 35 females, four males; *M*_age_ = 38.13 years, SD = 5.03; DLD group: 17 females, one male; *M*_age_ = 38.50 years, SD = 4.15; TLD group: 18 females, three males; *M*_age_ = 37.80 years, SD = 5.79) recruited through (*name of institution blinded for review*) mailing list and associated social media along with communication regarding the research study to speech pathology practices from major metropolitan centres whose details were accessible on the Speech Pathology Australia website. Participants were parents of children aged 4 to 12 years (total sample: 24 males, 15 females; *M*_age_ = 7.64 years, SD = 2.31; DLD group: 11 males, seven females; *M*_age_ = 7.37 years, SD = 2.50; TLD group; 13 males, eight females; *M*_age_ = 7.96‐years, SD = 2.10). This age range is appropriate as school‐aged children's participation in OESAs typically requires parental support and involvement. Furthermore, children in Australia generally begin formal schooling at age four and complete primary school by approximately age 12.

A total of 81 responses were received for this study. Parents reported the details of their child's diagnostic status. In this study, the TLD group refers to children who had no reported clinical diagnoses and no diagnoses specifically related to language development. Further, to align with the diagnostic guidelines outlined by the CATLISE consortium (Bishop et al. 2017), the following were excluded: parents of children with an autism diagnosis (*n* = 19; 12 of whom reported co‐occurring diagnoses of autism and DLD), parents of children with diagnoses other than DLD and autism (*n* = 22), and children with DLD whose parents reported demographic and diagnostic information but did not complete the OESA section of the study (*n* = 2). Several parents included in the final DLD group reported that their children had a co‐occurring ADHD diagnosis (*n* = 4). The final sample comprised primarily White, English‐speaking, cisgender, and educated Australian citizens. Further demographics are available in Tables S1 and S2 of the Supplementary Materials.

### 2.2 Procedure and Materials

The present study used an established OESA participation and experience online self‐report measure completed by a child's parent (see Farrell et al. 2024). Participants provided informed consent and were free to discontinue their participation at any point without penalty. Upon completing the study, they received a written debrief. Parents were first asked to provide demographic information regarding themselves and their child, including a diagnosis date and details about the diagnosing professional for those participants answering about a child diagnosed with DLD. Following this, participants were presented with an OESA definition and subsequently answered questions regarding levels of participation, which were presented in a fixed order.

To support understanding of the structure and practical administration of the measure, we briefly outline its components here. After the demographic section, parents responded to items across four domains: (1) ability and behaviour, (2) programme features, (3) environmental factors, and (4) social dynamics. Each item asked parents to rate how much the factor influenced their child's participation in a given OESA using a continuous sliding scale from negative (a barrier) to positive (a facilitator). These four domain question sets were repeated at each of the three levels of participation (fully engaged, partially engaged, and considered engagement). To further support interpretation of results, a full copy of the measure, including all items, is available here: (*link blinded for review*).

1. The four components of the ICF‐CY inform the content of the items within the measure: (1) the body, (2) activities and participation, (3) environmental factors, and (4) personal factors. These components align with the content of the questions presented to participants as follows (additionally, see Figure 1):
2. The body: This component informs items in the ability and behaviour domain, which assess how a child's physical, communicative, cognitive, and behavioural abilities influence their participation and experience.
3. Activities and participation: This component informs the three levels of participation. Items in the programme domain assess how features of the OESA, such as activity flexibility, behavioural accommodation, and parental support, affect a child's participation and experience.
4. Environmental factors: This component informs the environmental domain, which captures the impact of the physical environment (e.g., infrastructure and accessibility) on participation. It also informs the social domain, which examines the influence of social factors, including the attitudes and beliefs of others and the roles individuals play within the OESA's social environment.
5. Personal factors: This component is represented by the demographic information provided by participants.

To gain a comprehensive understanding of OESA participation and experience, we examined facilitators and barriers to participation across three levels:

1. Level One (fully engaged) asked parents to reflect on OESAs that their child participated in regularly during the past year.
2. Level Two (partially engaged) asks parents to comment on OESAs their child participated in but withdrew from over the past 5 years.
3. Level Three (considered engagement) asks parents to comment on OESAs that were contemplated but for which they have not actually participated over the past 5 years.

Although children may engage in an OESA for several years, Level One was limited to a 1‐year timeframe to enhance the reliability of recall. Hence, at Level One, the focus on recent experiences ensures accurate reporting while enabling parents to provide valuable insights into their child's OESA participation and experience. In contrast, Levels Two and Three use a 5‐year timeframe, recognising that parents may not have withdrawn their child from, or considered participation in, a given OESA within a single year. Extending the recall period to 5 years provides greater access to information about disengagement and barriers to participation.

Parents could list no OESAs if a level of participation was not applicable, or up to five OESAs if it was. Level One (fully engaged) included three open‐ended items. The first asked for the name of the OESA the child participated in, the second requested the average number of hours per week spent on the activity, and the third asked for the duration of participation in weeks. Level Two included two questions: the first asked for the name of the OESA the child participated in, and the second question specifically asked for the duration of participation prior to termination. Level Three (considered engagement) included a single open‐ended item asking participants to list the name of an OESA they had considered for their child but ultimately did not engage in.

For each level of OESA participation, the first OESA listed was designated as the reference for the following domain‐related items. Parents were then presented with 29 fixed‐order items and asked to rate the extent to which various factors influenced participation in the nominated OESA. The items were designed to capture information about the child's experiences within the specified OESA and whether these experiences functioned as barriers or facilitators to participation. The ability and behaviour domain consisted of eight items (e.g., *my child's behaviour*), the programme domain included seven items (e.g., *opportunities to start at beginner or introductory levels*), the environmental domain included six items (e.g., *the availability of transport)*, and the social domain included eight items (e.g., *attitudes of other parents towards my child*). These items were answered on a slider bar, which provided a continuous measure of experience as negative, neutral or positive. Scores are derived from the sliding scale and range from −1.00 (*a negative influence/barrier*) to 1.00 (*a positive influence/facilitator*), with 0.00 representing a neutral influence. Therefore, when a participant positioned the slider at any point of the bar, a continuous output score ranging from −1.00 to 1.00 was calculated.

### 2.3 OESA Categorisation

Each reported OESA was assigned to one of five mutually exclusive categories: religious, academic, the arts, sports, and community/service (see Farrell et al. 2024). The primary nature of the activity informed the assignment of a category for each of the OESAs listed by participants. The lead author and a research assistant independently categorised all OESAs reported by participants. The resulting interrater reliability for OESA category coding was 97.16%, and the four disagreements were resolved by discussion.

### 2.4 Measurement

The present study measured the breadth, total number, and intensity of OESA participation and the extent to which each domain acted as a barrier or facilitator to participation.

### 2.5 OESA Breadth Score

Each participant was assigned an OESA breadth score for each level of participation. This score was determined by counting the number of distinct OESA categories in which the participant engaged at each level.

### 2.6 OESA Total Score

Parents could report between one and five OESAs at each level of participation. An OESA total score was calculated for each level by summing the number of activities listed.

### 2.7 OESA Intensity Score

At Level One of participation (fully engaged), an OESA intensity score was calculated for each participant by multiplying the total number of hours per week by the total weeks of participation for each listed OESA. This score reflected the intensity of OESA participation within a 1‐year timeframe.

### 2.8 OESA Domain Scores

At each level of participation, participants rated 29 items representing four domains (i.e., ability and behaviour, programme, environmental, and social). Ratings were based on the first listed OESA and were scored using a sliding scale from −1.00 (*negative influence/barrier*) to 1.00 (*positive influence/facilitator*), with a score of 0.00 indicating a neutral influence on participation. Item scores within each domain were summed to generate four domain composite scores for each level of participation.

## 3 Results

### 3.1 Nature of the Data and Preliminary Analyses

Participants were only required to report on a level of participation if it was relevant to their experience. Due to varying sample sizes across levels (see Table S3 of the Supplementary Materials), analyses were conducted per level of participation.

The DLD and TLD groups were considered separately for preliminary analysis based on evidence that general participation could differ between groups (e.g., Lloyd‐Esenkaya et al. 2020; van der Niet et al. 2014).

Across all levels of participation, no significant differences in child age (all *p* ≥ 0.254) or sex (all *p* ≥ 0.435) were observed between the DLD and TLD groups; therefore, child age and sex were not considered further. A series of chi‐square tests revealed no significant differences between the DLD and TLD groups regarding the categories of OESAs (religious, academic, the arts, sports, and community/service) reported on at any level of participation (all *p* ≥ 0.206). Across both groups at all three levels of participation, the sports category was the most reported.

A series of independent samples *t*‐tests were performed to investigate whether the DLD and TDL groups differed on OESA breadth, the total number of reported OESAs, OESA intensity (at Level One of participation, fully engaged), and OESA experience domains across three levels of participation. Where a significant difference between groups for an OESA domain was found, subsequent independent samples *t*‐tests were conducted to examine differences at the item level within that domain. It should be noted that while Bonferroni corrections may reduce Type I error risk, they can be overly conservative, especially with correlated comparisons. Therefore, we encourage readers to consider both statistical significance and effect sizes when interpreting the results reported here.

### 3.2 OESA Participation and Experiences

#### 3.2.1 Level One of Participation

At Level One (fully engaged), the DLD group did not differ significantly from the TLD group regarding OESA intensity, the total number of OESAs or the breadth of OESAs that were participated in. Further, the DLD group did not differ significantly from the TLD group within the ability and behaviour domain, the program domain, the environmental domain, and the social domain (see Table 1). In summary, parental reports indicated that children with DLD and those with TLD participated in a similar number of OESAs, across comparable categories, for a similar number of hours on average per week. From a parental perspective, domain ratings indicated that the child's ability and behaviour, features of the OESA, and the social environment the OESA took place in were positive facilitators of OESA participation for children with DLD and their TLD peers.

**TABLE 1:** Statistics of independent samples *t*‐tests at Level One (fully engaged) of participation.

	DLD		TLD					
Variable	*Μ*	SD	*Μ*	SD	*t*	*p*	*d*	95 % CI for *d*
OESA intensity	135.06	120.54	90.33	85.31	−1.32	0.195	−0.44	[−1.09, 0.22]
OESA total score	2.06	1.12	2.00	1.23	−0.16	0.874	0.33	[−0.70, 0.60]
OESA breadth	1.38	0.50	1.19	0.40	−1.24	0.222	0.34	[−1.07, 0.25]
Ability and behaviour domain	3.40	4.04	5.18	2.00	1.76	0.088	0.58	[−0.09, 1.24]
Programme domain	3.20	2.30	3.63	1.73	0.65	0.517	0.22	[−0.44, 0.87]
Environmental domain	1.88	1.86	2.81	1.52	1.67	0.104	0.55	[−0.11, 1.21]
Social domain	2.32	2.43	2.90	1.52	0.74	0.464	0.25	[−0.41, 0.90]

*Note*: All degrees of freedom (df) = 35.

Abbreviations: DLD, developmental language disorder; TLD, typical language developing.

#### 3.2.2 Level Two of Participation

At Level Two (partially engaged), the DLD group did not differ significantly from the TLD group regarding the total number of OESAs participated in (see Table 2). Regarding OESA breadth scores, all participants at Level Two received a breadth score of 1. Hence, no statistical tests were run regarding this variable. Therefore, both groups participated in and withdrew from a similar number and range of OESAs. Regarding factors that were perceived to influence participation, the DLD group differed significantly from the TLD group within the ability and behaviour domain at Level Two. Although both groups reported this domain as a facilitator of OESA participation, the DLD group reported it as less positive than the TLD group. In contrast, groups did not differ significantly regarding the programme, social, or environmental domains. From a parental perspective, domain‐level ratings suggested that children's ability and behaviour were perceived as less supportive of sustained OESA participation for children with DLD compared to their TLD peers.

**TABLE 2:** Statistics of independent samples *t*‐tests at Level Two (partially engaged) of participation.

	DLD		TLD					
Variable	*Μ*	SD	*Μ*	SD	*t*	*p*	*d*	95 % CI for *d*
OESA total score	1.39	0.77	1.18	0.41	−0.79	0.440	−0.32	[−1.13, 0.49]
**Ability and behaviour Domain**	**0.33**	**3.52**	**3.52**	**3.23**	**2.92**	**0.032**	**0.94**	**[**−**0.34, 1.29]**
Programme domain	0.90	2.50	2.62	2.27	1.75	0.094	0.72	[−0.12, 1.54]
Environmental domain	1.41	1.33	1.93	1.92	0.78	0.441	0.32	[−0.49, 1.13]
Social domain	0.89	3.12	2.29	2.58	1.18	0.251	0.48	[−0.34, 1.29]

*Note*: All degrees of freedom (df) = 22; bolded values indicate significance at *p* < 0.05.

Abbreviations: DLD, developmental language disorder; TLD, typical language developing.

#### 3.2.3 Level Three of Participation

At Level Three (considered engagement), the DLD and TLD groups did not differ significantly regarding the total number or breadth of OESAs considered by parents (see Table 3). These findings indicate that both groups considered a similar number and a similar breadth of OESAs for their child to participate in. Further, the groups did not significantly differ in the ability and behaviour, programme, environmental, or social domains (see Table 3). Collectively, domain ratings indicated that from a parental perspective, the child's ability and behaviour, features of an OESA, and the social environment the OESA may take place in were similarly perceived for both children with DLD and their TLD peers when considering participation in novel OESAs.

**TABLE 3:** Statistics of independent samples *t*‐tests at Level Three (considered engagement) of participation.

	DLD		TLD					
Variable	*Μ*	SD	*Μ*	SD	*t*	*p*	*d*	95 % CI for *d*
OESA total score	1.75	0.89	1.27	0.46	−1.74	0.096	−0.76	[−1.64, 0.13]
OESA breadth	1.25	0.46	1.13	0.35	−0.68	0.504	−0.30	[−1.16, 0.57]
Ability and behaviour domain	0.83	3.45	2.88	2.79	1.55	0.135	0.68	[−0.21, 1.55]
Programme domain	0.30	1.63	1.53	2.54	1.23	0.233	0.54	[−0.34, 1.41]
Environmental domain	0.40	1.11	0.75	2.16	0.43	0.427	0.19	[−0.68, 1.05]
Social domain	−0.04	1.99	1.78	2.23	1.94	0.067	0.85	[−0.06, 1.73]

*Note*: All degrees of freedom (df) = 21.

Abbreviations: DLD, developmental language disorder; TLD, typical language developing.

### 3.3 OESA Domain Follow‐Up Analyses

#### 3.3.1 Level Two of Participation

The ability and behaviour domain presented as significantly different between groups in OESAs that families ultimately chose to withdraw from (see Table 2). As shown in Table 4, items 1 through 4 significantly differed between groups. Children's motor and social skills and coordination were reported as less positive facilitators of OESA participation for children with DLD compared to their TLD peers. Further, for children with DLD, their communication skills were perceived by parents as a barrier to OESA participation, and this was significantly different from TLD children.

**TABLE 4:** Mean scores for the ability and behaviour domain at Level Two of OESA participation.

	DLD	TLD				
Item	*Μ* (SD)	*Μ* (SD)	*t*	*p*	*d*	95 % CI for *d*
**1. My child's communication skills**	**−0.15 (0.59)**	**0.36 (0.54)**	**2.17**	**0.041**	**0.57**	**[0.36, 1.73]**
**2. My child's motor skills**	**0.01 (0.70)**	**0.81 (0.27)**	**3.55**	**0.002**	**0.55**	**[0.53, 2.35]**
**3. My child's social skills**	**0.09 (0.61)**	**0.60 (0.39)**	**2.37**	**0.027**	**0.52**	**[0.11, 1.82]**
**4. My child's coordination**	**0.09 (0.68)**	**0.70 (0.38)**	**2.67**	**0.014**	**0.56**	**[0.22, 1.95]**
5. My child's attention	−0.03 (0.56)	0.24 (0.69)	1.05	0.307	0.62	[−0.39, 1.24]
6. My child's interests	0.19 (0.49)	0.33 (0.68)	0.57	0.578	0.59	[−0.58, 1.05]
7. My child's behaviour	0.13 (0.48)	0.24 (0.63)	0.49	0.632	0.56	[−0.61, 1.00]
8. My child's sensory preferences	0.01 (0.45)	0.25 (0.60)	1.12	0.274	0.53	[−0.36, 1.27]

*Note*: All degrees of freedom (df) = 22; bolded values indicate significance at *p* < 0.05.

Abbreviations: DLD, developmental language disorder; TLD, typical language developing.

## 4 Discussion

The present study offers novel evidence of OESA participation and experiences for children with DLD compared to their TLD peers. By applying the ICF‐CY framework, we captured a multidimensional perspective on participation, incorporating children's abilities and behaviours and the environmental, programme, and social factors influencing their experiences. This approach allowed us to assess participation across three levels—fully engaged, partially engaged, and considered engagement—providing insights beyond a simple comparison of participation rates. Our findings indicate that, overall, children with DLD participate in OESAs at levels comparable to their TLD peers and generally have positive experiences. However, when disengagement from an OESA occurs, ability‐related factors, such as communication skills, motor skills, social skills, and coordination, play a more significant role in shaping participation for children with DLD. By identifying facilitators and barriers to various participation thresholds, this study highlights the importance of fostering supportive environments that maximise the developmental benefits of OESAs for children with DLD.

### 4.1 OESA Participation and Experience

Overall, children with DLD participated in OESAs at comparable rates of breadth, intensity and total number to their TLD peers, and parents reported largely positive facilitative experiences across all three participation levels (fully engaged, partially engaged, and considered engagement). Therefore, these findings suggest that, despite the linguistic and social challenges often associated with a DLD diagnosis, OESAs may offer accessible and meaningful opportunities for participation. This is supported by the finding that children with DLD participated in a comparable number of OESAs and for a similar duration each week as their TLD peers, and by broadly similar parent ratings of participation facilitators across groups. Together, these indicators suggest that many children with DLD can access the developmental benefits of such participation (Bohnert et al. 2019; May et al. 2021; McCabe et al. 2016; Peck et al. 2008). This finding is important as it provides novel insights into OESA participation rates for children with DLD in Australia, highlighting that for this sample at least, previous findings regarding children with developmental disabilities may not always generalise to this population (Engel‐Yeger et al. 2009; Farrell et al. 2024; King et al. 2010; Law et al. 2011).

However, when disengagement did occur, ability‐ and behaviour‐related factors emerged as important influences. Specifically, a child's motor skills, social skills, and coordination were found to be less positive facilitators at Level Two (partially engaged) of participation for children with DLD compared to their TLD peers. Further, at the same level, a child's communication skills were reported as a barrier to participation for children with DLD, while it remained as a facilitator for the TLD group. This suggests that, although not always perceived as outright barriers, these factors may subtly shape the quality of participation over time, echoing findings from broader disability research that highlight how participation can be constrained by skill demands even when access is technically available (King et al. 2010; Law et al. 2011). Importantly, the findings indicate that rather than facing widespread barriers to OESAs, children with DLD may encounter specific challenges that influence their engagement in more nuanced ways. Future research should explore whether these factors accumulate over time to influence longer‐term participation patterns, even when not immediately limiting.

Despite these challenges, the generally high levels of participation and limited reports of perceived barriers in this study suggest that OESAs may serve as a relatively accessible and positive context for children with DLD. Further, given that children with DLD are at greater risk of social difficulties (Wieczorek et al. 2024), OESAs may provide a valuable environment where they can develop friendships, build social confidence, and practice key interpersonal skills in a setting that is often less linguistically demanding than academic contexts (McGregor et al. 2023; Toseeb and St Clair 2020). As such, maintaining and supporting participation in OESAs may have long‐term benefits for immediate social engagement and broader developmental outcomes.

In sum, while OESA participation appears largely accessible and positive for children with DLD, understanding and addressing ability‐ and behaviour‐related challenges is key to ensuring sustained engagement. These findings challenge deficit‐based perspectives that assume participation difficulties are inevitable for children with DLD, instead demonstrating that in the right contexts and with the right support, children with DLD may access and benefit from OESAs in ways similar to their TLD peers (Huang et al. 2020; May et al. 2021).

### 4.2 Limitations and Future Directions

While this study offers valuable insights into the participation and experiences of children with DLD in OESAs, several limitations must be acknowledged. First, the small sample size of children with DLD, though comparable to previous studies (Aguilar‐Mediavilla et al. 2019; Fyfe et al. 2019; Lloyd‐Esenkaya et al. 2020; van der Niet et al. 2014), was constrained by adherence to recommended diagnostic criteria, which recommends exclusion when language disorder occurs in the presence of ASD and other genetic diagnoses (Bishop et al. 2017). This decision was made to align with the study's aims—focusing specifically on the OESA participation and unique experiences of children with DLD to establish a foundation for future research. Furthermore, recruiting participants was extremely challenging despite outreach through clinicians, organisations, social media, databases, and researcher networks. This difficulty likely reflects poor identification of DLD in the community and the broader issue of low public awareness of DLD (see Kim et al. 2022; McGregor et al. 2020). Future studies should continue to explore effective recruitment strategies while acknowledging the need for greater community awareness of DLD.

Second, the present study relied on parental reports requiring retrospective recall of their child's participation in OESAs, with some reports spanning up to 5 years. This approach may introduce recall bias, leading to possible over‐ or under‐reporting of participation and experiences. Additionally, as with any opt‐in recruitment, there is a risk of self‐selection bias, where parents with particularly strong or positive views may have been more likely to participate. Observational data collection would enhance accuracy in future studies. Moreover, our cross‐sectional design limits conclusions about changes in participation and experiences over time. Future longitudinal research with a naturalistic observational approach is needed to examine how participation in OESAs evolves as children with DLD develop, particularly regarding continued OESA engagement and potential long‐term benefits. Also, missing is the perspective of children with DLD, whose experience with OESAs may not completely align with that of their parents.

Third, we did not assess children's language capabilities. However, prior research suggests that the severity of language impairment is not necessarily a strong predictor of language‐related function or support needs (McGregor et al. 2020). Future studies may explore whether specific language profiles or the presence of comorbid difficulties (e.g., ADHD, motor coordination difficulties) influence OESA participation differently.

Fourth, our results indicate that OESA participation is mainly positive for children with DLD. However, future research should explore how these activities protect against challenges that arise later in life. Individuals with DLD are at increased risk of poorer mental health outcomes (Yew and O'Kearney 2013), and structured extracurricular activities may offer valuable opportunities for social connection and emotional well‐being. Longitudinal studies could assess whether early engagement in OESAs mitigates later social or mental health difficulties.

Lastly, our sample predominantly consisted of children from higher socioeconomic status (SES) backgrounds, limiting generalisability to more socioeconomically diverse populations. Future studies should explore whether children with DLD from lower SES backgrounds have different participation experiences compared to their higher SES peers and whether financial or structural barriers impact participation in and access to OESAs.

### 4.3 Practical Implications

The findings presented here suggest that OESAs may provide valuable opportunities for socialisation, skill development, and overall well‐being for children with DLD. However, ensuring these positive experiences are sustained requires targeted support at policy and programme levels.

One important implication is the need for policy‐level investment in making OESAs more accessible and supportive for children with DLD. Given the developmental benefits associated with participation, increased funding could be directed towards reducing financial barriers, expanding inclusive programming, and providing training for OESA facilitators. By strengthening accessibility and awareness, these activities can continue to serve as enriching spaces where children with DLD can build friendships and gain confidence in their social interactions.

At a programme level, findings suggest that while ability‐ and behaviour‐related challenges may sometimes lead to disengagement, they do not appear to prevent participation entirely. Rather than requiring major structural adaptations, targeted support strategies could help sustain engagement and improve experiences. Collaboration between allied health professionals, families, and OESA facilitators may be valuable in ensuring that children with DLD receive appropriate support. Training facilitators on simple, developmentally appropriate strategies, fostering peer support networks, and creating a more inclusive environment could address the subtle barriers identified in this study.

### 4.4 Conclusion

The present study provides novel insights into the participation and experiences of children with DLD in OESAs, demonstrating that their involvement is largely comparable to that of their TLD peers. While ability‐related factors may present challenges, these do not preclude participation but rather shape engagement in nuanced ways. These findings highlight the potential of OESAs as developmentally rich environments that can support the development of children with DLD and, more broadly, promote positive experiences for all children, regardless of ability.

## Ethics Statement

Approval was obtained from the Low and Negligible Risk Ethics Sub‐Committee of The University of Queensland (Approval Number: 2020000981).

## Conflicts of Interest

The authors declare no conflicts of interest.

## Supporting information



Supplementary Information

Supplementary Information

## Data Availability

The data that support the findings of this study are available from the corresponding author upon reasonable request.
