# Supplementary material for: Participation and Experiences in Extracurricular Activities for Children With Developmental Language Disorder and Their Peers
Source: Int J Lang Commun Disord. 2025 Oct 5;60(6):e70134. doi: 10.1111/1460-6984.70134 (PMC12497680; doi:10.1111/1460-6984.70134)
Supplement: Supplementary file 1 — Supplementary Information [file JLCD-60-0-s002.docx]

**Supplementary Materials**

**Table S1**

*Sociodemographic Characteristics of Participants (Parents)*

| Characteristic | Full Sample | |
| --- | --- | --- |
|  | *n* | % |
| **Annual Household Income ($AUD before taxation)**  Under 30,000  31,000-50,000  51,000-75,000  76,000-99,000  100,000 or over  Preferred not to respond | 5  5  8  6  14  1 | 12.82  12.82  20.51  15.39  35.90  2.56 |
| **Primary Language**  English  Other | 36  3 | 92.31  7.69 |
| **Nationality**  Australian  Other | 37  2 | 94.87  5.13 |
| **Race**  Black  White  Asian  Aboriginal Australian  Biracial  Multiracial | 2  31  3  1  1  1 | 5.14  79.49  7.69  2.56  2.56  2.56 |
| **Education**  Less than Year 10  Year 12 or equivalent  Trade/Apprentice  Certificate/Diploma  University Degree  Higher University Degree | 1  2  2  11  11  12 | 0.50  35.80  1.70  13.10  26.90  19.20 |

*Note.* *N* = 39.

**Table S2**

*Sociodemographic Characteristics of Children Represented*

| Characteristic | Full Sample | | DLD | | TLD | |
| --- | --- | --- | --- | --- | --- | --- |
|  | *n* | % | *n* | % | *n* | % |
| **Child Assigned Sex**  Male  Female | 24  15 | 61.54  38.46 | 11  7 | 61.11  38.89 | 13  8 | 61.91  38.09 |
| **Nationality**  Australian  Other | 36  3 | 92.31  7.69 | 16  2 | 88.89  11.11 | 20  1 | 95.24  4.76 |
| **Race**  Black  White  Asian  Aboriginal Australian  Biracial  Multiracial | 2  29  3  1  2  2 | 5.13  74.36  7.69  2.56  5.13  5.13 | 2  13  2  -  -  1 | 11.11  72.22  11.11  -  -  5.56 | -  16  1  1  1  2 | -  76.19  4.76  4.76  4.76  9.52 |
|  |  | |  | |  | |

*Note.* *N* = 39, DLD *n* = 18, TLD *n* = 21.

**Table S3**

*The Final Sample of Participants for Each Group at Each Level of Participation*

|  | Level of Participation | | |
| --- | --- | --- | --- |
|  | Level One | Level Two | Level Three |
| DLD | 16 | 13 | 8 |
| TLD | 21 | 11 | 15 |
| Total | 37 | 24 | 23 |

*Note.* Level One = fully engaged, Level Two = partially engaged, Level Three = considered engagement,

DLD = developmental language disorder, TLD = typical language developing.
