# Supplementary material for: Participation and Experiences in Extracurricular Activities for Children With Developmental Language Disorder and Their Peers
Source: Int J Lang Commun Disord. 2025 Oct 5;60(6):e70134. doi: 10.1111/1460-6984.70134 (PMC12497680; doi:10.1111/1460-6984.70134)
Supplement: Supplementary file 2 — Supplementary Information [file JLCD-60-0-s001.docx]

DLD OESA 2024

***Insert PIS/Consent***

**The following questions aim to gather information relating to you**

**What is your current postcode?**

**What is your current age in years? (please enter numbers e.g., 44)**

**What is your sex**

- Male
- Female
- A term not listed above - please specify __________________________________________________

**What is your country of primary citizenship?**

- Australia
- Other (please list) __________________________________________________

**Please select the option that best describes your race**

- Aboriginal Australian
- Torres Strait Islander
- Middle Eastern
- Pacific Islander
- Black
- Asian
- LatinX
- White
- Biracial
- Multiracial
- Other (please list) __________________________________________________
- Prefer not to respond

**What is your primary language (the language you speak most often)?**

- English
- Other (please list) __________________________________________________

**What is your personal annual income before taxation ($AUD)?**

- $30,000 or less
- $31,000 – $50,000
- $51,000 – $75,000
- $76,000 – $99,000
- $100,000 or more
- Prefer not to respond

**What is your highest level of education?**

- Less than Year 10
- Year 10 or equivalent (e.g., School Certificate)
- Year 12 or equivalent (e.g., Higher School Certificate)
- Trade/apprenticeship
- Certificate/diploma
- University degree
- Higher university degree (e.g., Grad Dip, Masters, PhD)
- Prefer not to respond

End of Block: Parent Demographics

Start of Block: Child Demographics

**Please answer the following questions in regard to your child. *If you have more than one child, please select only one (between the ages of 4 and 12 years old) for the entirety of this questionnaire.***

**Has your child received any of these clinical diagnoses? *please select all that apply, if none apply please select none at the bottom of this list***

- Autism (ASD)
- ADHD
- A Developmental Language Disorder (e.g., DLD or SLI)
- Global Developmental Delay (GDD)
- Deaf or Hard of Hearing
- An Intellectual Disability
- A Genetic Condition (please give details in the box below) __________________________________________________
- Other (please give details in the box below) __________________________________________________
- None

What is the name and occupation (e.g. Psychologist) of the individual who made the diagnosis

- Name of the individual __________________________________________________
- Their occupation __________________________________________________

What was the date of your child's diagnosis (DD/MM/YYYY)

________________________________________________________________

**What is your child’s current age in years and months**

- Years __________________________________________________
- Months __________________________________________________

**What is your child’s sex**

- Male
- Female
- A term not listed above - please specify __________________________________________________

**What is your child's country of primary citizenship?**

- Australia
- Other (please list) __________________________________________________

**Please select the option that best describes your child's race**

- Aboriginal Australian
- Torres Strait Islander
- Middle Eastern
- Pacific Islander
- Black
- Asian
- LatinX
- White
- Biracial
- Multiracial
- Other (please list) __________________________________________________
- Prefer not to respond

End of Block: Child Demographics

Start of Block: OESA Definition

Organised extracurricular social activities are those that take place with other individuals, peers or friends outside of school hours. They are formally organised and structured in nature. They may be supervised by an adult, coach or facilitator. They are activities that take place regularly, maybe weekly and for an extended amount of time, such as a season or term. They are those activities that provide opportunities to connect with others and develop skills that relate to the activity. It may be helpful to think about these activities in the following five categories; Sports (e.g., soccer, cricket, netball), The Arts (e.g., dance classes, choir, bands), Academic (e.g., chess club, debating), Community (e.g., Scouts, Girl Guides, gardening groups), and Religious (e.g., youth group, Sunday school).

End of Block: OESA Definition

Start of Block: L1: Participated In

Please answer the following questions in regards to organised extracurricular social activities your child maintained regular participation in during the **last 12 months**

***We will ask you some follow up questions about the first activity listed***

*If no activity is relevant there is an option at the bottom of this page*

- Please list the name of one activity your child maintained regular participation in during the last 12 months   __________________________________________________
- In the last 12 months, on average, how many hours of this activity did your child complete per week?   __________________________________________________
- How many weeks or months in the last 12 months did your child participate in this activity?   __________________________________________________

OR

- My child did not maintain regular participation in any organised extracurricular social activities during the last 12 months

Add another organised extracurricular social activity your child maintained regular participation in during the **last 12 months**

- Yes
- No

End of Block: L1: Participated In

Start of Block: L1 Behaviour

To what extent do you feel each of the following factors influenced participation in ${L1 A/ChoiceTextEntryValue/1}:

| 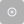 |
| --- |

My child’s communication skills

|  | **Negative Influence** | **Did Not Influence** | **Positive Influence** | Not Applicable |
| --- | --- | --- | --- | --- |

|  | 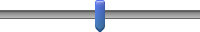 |
| --- | --- |

| 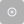 |
| --- |

My child’s motor skills

|  | **Negative Influence** | **Did Not Influence** | **Positive Influence** | Not Applicable |
| --- | --- | --- | --- | --- |

|  | 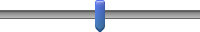 |
| --- | --- |

| 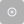 |
| --- |

My child’s social skills

|  | **Negative Influence** | **Did Not Influence** | **Positive Influence** | Not Applicable |
| --- | --- | --- | --- | --- |

|  | 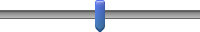 |
| --- | --- |

| 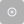 |
| --- |

My child’s coordination

|  | **Negative Influence** | **Did Not Influence** | **Positive Influence** | Not Applicable |
| --- | --- | --- | --- | --- |

|  | 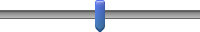 |
| --- | --- |

| 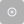 |
| --- |

My child’s attention while participating

|  | **Negative Influence** | **Did Not Influence** | **Positive Influence** | Not Applicable |
| --- | --- | --- | --- | --- |

|  | 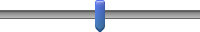 |
| --- | --- |

| 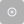 |
| --- |

My child’s interests

|  | **Negative Influence** | **Did Not Influence** | **Positive Influence** | Not Applicable |
| --- | --- | --- | --- | --- |

|  | 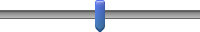 |
| --- | --- |

| 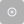 |
| --- |

My child’s behaviour

|  | **Negative Influence** | **Did Not Influence** | **Positive Influence** | Not Applicable |
| --- | --- | --- | --- | --- |

|  | 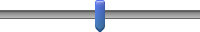 |
| --- | --- |

| 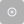 |
| --- |

My child’s sensory preferences

|  | **Negative Influence** | **Did Not Influence** | **Positive Influence** | Not Applicable |
| --- | --- | --- | --- | --- |

|  | 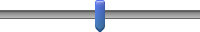 |
| --- | --- |

End of Block: L1 Behaviour

Start of Block: L1 Social

To what extent do you feel each of the following factors influenced participation in ${L1 A/ChoiceTextEntryValue/1}:

| 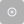 |
| --- |

Attitudes of other parents towards me

|  | **Negative Influence** | **Did Not Influence** | **Positive Influence** | Not Applicable |
| --- | --- | --- | --- | --- |

|  | 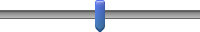 |
| --- | --- |

| 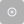 |
| --- |

Attitudes of facilitators or coaches towards me

|  | **Negative Influence** | **Did Not Influence** | **Positive Influence** | Not Applicable |
| --- | --- | --- | --- | --- |

|  | 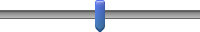 |
| --- | --- |

| 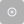 |
| --- |

Attitudes of other parents towards my child

|  | **Negative Influence** | **Did Not Influence** | **Positive Influence** | Not Applicable |
| --- | --- | --- | --- | --- |

|  | 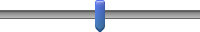 |
| --- | --- |

| 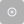 |
| --- |

Attitudes of facilitators or coaches towards my child

|  | **Negative Influence** | **Did Not Influence** | **Positive Influence** | Not Applicable |
| --- | --- | --- | --- | --- |

|  | 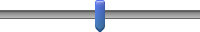 |
| --- | --- |

| 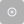 |
| --- |

The skill level of facilitators or coaches

|  | **Negative Influence** | **Did Not Influence** | **Positive Influence** | Not Applicable |
| --- | --- | --- | --- | --- |

|  | 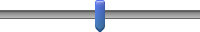 |
| --- | --- |

| 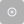 |
| --- |

The individual or group nature of the activity

|  | **Negative Influence** | **Did Not Influence** | **Positive Influence** | Not Applicable |
| --- | --- | --- | --- | --- |

|  | 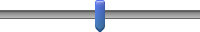 |
| --- | --- |

| 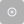 |
| --- |

My child’s possible exposure to bullying

|  | **Negative Influence** | **Did Not Influence** | **Positive Influence** | Not Applicable |
| --- | --- | --- | --- | --- |

|  | 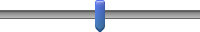 |
| --- | --- |

| 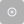 |
| --- |

My beliefs about the value of the activity for my child

|  | **Negative Influence** | **Did Not Influence** | **Positive Influence** | Not Applicable |
| --- | --- | --- | --- | --- |

|  | 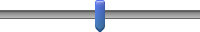 |
| --- | --- |

End of Block: L1 Social

Start of Block: L1 Environmental

To what extent do you feel each of the following factors influenced participation in ${L1 A/ChoiceTextEntryValue/1}:

| 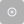 |
| --- |

The availability of appropriate and safe equipment and facilities

|  | **Negative Influence** | **Did Not Influence** | **Positive Influence** | Not Applicable |
| --- | --- | --- | --- | --- |

|  | 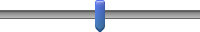 |
| --- | --- |

| 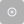 |
| --- |

The availability of transport

|  | **Negative Influence** | **Did Not Influence** | **Positive Influence** | Not Applicable |
| --- | --- | --- | --- | --- |

|  | 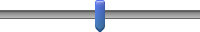 |
| --- | --- |

| 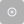 |
| --- |

My child’s familiarity with surrounds

|  | **Negative Influence** | **Did Not Influence** | **Positive Influence** | Not Applicable |
| --- | --- | --- | --- | --- |

|  | 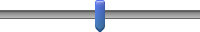 |
| --- | --- |

| 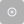 |
| --- |

Our family’s availability

|  | **Negative Influence** | **Did Not Influence** | **Positive Influence** | Not Applicable |
| --- | --- | --- | --- | --- |

|  | 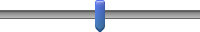 |
| --- | --- |

| 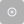 |
| --- |

The impact of my child’s desire for ‘screen time’ on their participation

|  | **Negative Influence** | **Did Not Influence** | **Positive Influence** | Not Applicable |
| --- | --- | --- | --- | --- |

|  | 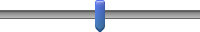 |
| --- | --- |

| 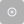 |
| --- |

The cost of the activity

|  | **Negative Influence** | **Did Not Influence** | **Positive Influence** | Not Applicable |
| --- | --- | --- | --- | --- |

|  | 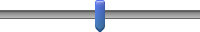 |
| --- | --- |

End of Block: L1 Environmental

Start of Block: L1 Program

To what extent do you feel each of the following factors influenced participation in ${L1 A/ChoiceTextEntryValue/1}:

| 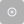 |
| --- |

This activity’s accommodation of my child’s behaviour

|  | **Negative Influence** | **Did Not Influence** | **Positive Influence** | Not Applicable |
| --- | --- | --- | --- | --- |

|  | 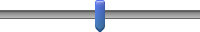 |
| --- | --- |

| 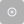 |
| --- |

How the activity makes my child feel

|  | **Negative Influence** | **Did Not Influence** | **Positive Influence** | Not Applicable |
| --- | --- | --- | --- | --- |

|  | 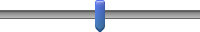 |
| --- | --- |

| 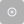 |
| --- |

Opportunities to start the activity at beginner or introductory levels

|  | **Negative Influence** | **Did Not Influence** | **Positive Influence** | Not Applicable |
| --- | --- | --- | --- | --- |

|  | 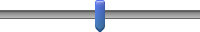 |
| --- | --- |

| 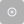 |
| --- |

The flexibility of the activity (e.g. relaxed rules)

|  | **Negative Influence** | **Did Not Influence** | **Positive Influence** | Not Applicable |
| --- | --- | --- | --- | --- |

|  | 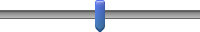 |
| --- | --- |

| 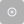 |
| --- |

The opportunity for social connection with peers, friends, parents or a buddy

|  | **Negative Influence** | **Did Not Influence** | **Positive Influence** | Not Applicable |
| --- | --- | --- | --- | --- |

|  | 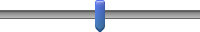 |
| --- | --- |

| 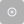 |
| --- |

The use of flexible and alternative communication styles

|  | **Negative Influence** | **Did Not Influence** | **Positive Influence** | Not Applicable |
| --- | --- | --- | --- | --- |

|  | 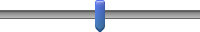 |
| --- | --- |

| 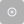 |
| --- |

My partner, co-parent or spouse’s support of my child’s involvement in the activity

|  | **Negative Influence** | **Did Not Influence** | **Positive Influence** | Not Applicable |
| --- | --- | --- | --- | --- |

|  | 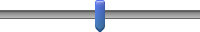 |
| --- | --- |

End of Block: L1 Program

Start of Block: L2: Withdrawn From

In the past five years what organised extracurricular social activities have you **involved your child in and then withdrawn them from**

***We will ask you some follow up questions about the first activity listed***

*If no activity is relevant there is an option at the bottom of this page*

- Please list the name of one activity your child was involved with   __________________________________________________
- On average how many hours of this activity did your child complete per week   __________________________________________________
- How long did your child participate in this activity   __________________________________________________

OR

- My child has not been involved in a organised extracurricular social activity in the past five years

Add another organised extracurricular social activity your child has been **involved in and withdrawn from in the past five years**

- Yes
- No

End of Block: L2: Withdrawn From

Start of Block: L2 Behaviour

To what extent do you feel each of the following factors influenced participation in ${L2 A/ChoiceTextEntryValue/1}:

| 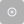 |
| --- |

My child’s communication skills

|  | **Negative Influence** | **Did Not Influence** | **Positive Influence** | Not Applicable |
| --- | --- | --- | --- | --- |

|  | 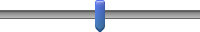 |
| --- | --- |

| 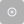 |
| --- |

My child’s motor skills

|  | **Negative Influence** | **Did Not Influence** | **Positive Influence** | Not Applicable |
| --- | --- | --- | --- | --- |

|  | 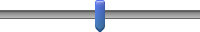 |
| --- | --- |

| 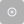 |
| --- |

My child’s social skills

|  | **Negative Influence** | **Did Not Influence** | **Positive Influence** | Not Applicable |
| --- | --- | --- | --- | --- |

|  | 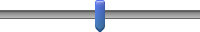 |
| --- | --- |

| 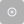 |
| --- |

My child’s coordination

|  | **Negative Influence** | **Did Not Influence** | **Positive Influence** | Not Applicable |
| --- | --- | --- | --- | --- |

|  | 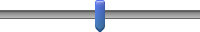 |
| --- | --- |

| 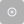 |
| --- |

My child’s attention while participating

|  | **Negative Influence** | **Did Not Influence** | **Positive Influence** | Not Applicable |
| --- | --- | --- | --- | --- |

|  | 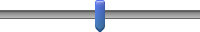 |
| --- | --- |

| 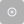 |
| --- |

My child’s interests

|  | **Negative Influence** | **Did Not Influence** | **Positive Influence** | Not Applicable |
| --- | --- | --- | --- | --- |

|  | 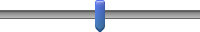 |
| --- | --- |

| 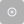 |
| --- |

My child’s behaviour

|  | **Negative Influence** | **Did Not Influence** | **Positive Influence** | Not Applicable |
| --- | --- | --- | --- | --- |

|  | 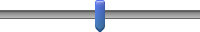 |
| --- | --- |

| 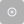 |
| --- |

My child’s sensory preferences

|  | **Negative Influence** | **Did Not Influence** | **Positive Influence** | Not Applicable |
| --- | --- | --- | --- | --- |

|  | 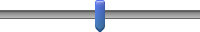 |
| --- | --- |

End of Block: L2 Behaviour

Start of Block: L2 Social

To what extent do you feel each of the following factors influenced participation in ${L2 A/ChoiceTextEntryValue/1}:

| 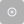 |
| --- |

Attitudes of other parents towards me

|  | **Negative Influence** | **Did Not Influence** | **Positive Influence** | Not Applicable |
| --- | --- | --- | --- | --- |

|  | 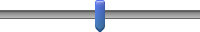 |
| --- | --- |

| 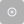 |
| --- |

Attitudes of facilitators or coaches towards me

|  | **Negative Influence** | **Did Not Influence** | **Positive Influence** | Not Applicable |
| --- | --- | --- | --- | --- |

|  | 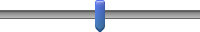 |
| --- | --- |

| 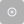 |
| --- |

Attitudes of other parents towards my child

|  | **Negative Influence** | **Did Not Influence** | **Positive Influence** | Not Applicable |
| --- | --- | --- | --- | --- |

|  | 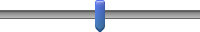 |
| --- | --- |

| 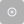 |
| --- |

Attitudes of facilitators or coaches towards my child

|  | **Negative Influence** | **Did Not Influence** | **Positive Influence** | Not Applicable |
| --- | --- | --- | --- | --- |

|  | 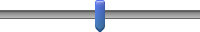 |
| --- | --- |

| 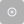 |
| --- |

The skill level of facilitators or coaches

|  | **Negative Influence** | **Did Not Influence** | **Positive Influence** | Not Applicable |
| --- | --- | --- | --- | --- |

|  | 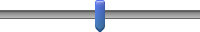 |
| --- | --- |

| 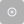 |
| --- |

The individual or group nature of the activity

|  | **Negative Influence** | **Did Not Influence** | **Positive Influence** | Not Applicable |
| --- | --- | --- | --- | --- |

|  | 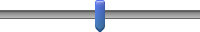 |
| --- | --- |

| 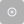 |
| --- |

My child’s possible exposure to bullying

|  | **Negative Influence** | **Did Not Influence** | **Positive Influence** | Not Applicable |
| --- | --- | --- | --- | --- |

|  | 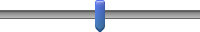 |
| --- | --- |

| 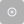 |
| --- |

My beliefs about the value of the activity for my child

|  | **Negative Influence** | **Did Not Influence** | **Positive Influence** | Not Applicable |
| --- | --- | --- | --- | --- |

|  | 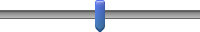 |
| --- | --- |

End of Block: L2 Social

Start of Block: L2 Environmental

To what extent do you feel each of the following factors influenced participation in ${L2 A/ChoiceTextEntryValue/1}:

| 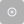 |
| --- |

The availability of appropriate and safe equipment and facilities

|  | **Negative Influence** | **Did Not Influence** | **Positive Influence** | Not Applicable |
| --- | --- | --- | --- | --- |

|  | 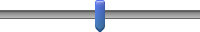 |
| --- | --- |

| 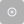 |
| --- |

The availability of transport

|  | **Negative Influence** | **Did Not Influence** | **Positive Influence** | Not Applicable |
| --- | --- | --- | --- | --- |

|  | 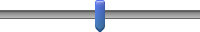 |
| --- | --- |

| 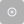 |
| --- |

My child’s familiarity with surrounds

|  | **Negative Influence** | **Did Not Influence** | **Positive Influence** | Not Applicable |
| --- | --- | --- | --- | --- |

|  | 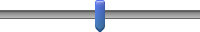 |
| --- | --- |

| 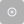 |
| --- |

Our family’s availability

|  | **Negative Influence** | **Did Not Influence** | **Positive Influence** | Not Applicable |
| --- | --- | --- | --- | --- |

|  | 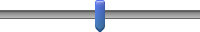 |
| --- | --- |

| 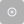 |
| --- |

The impact of my child’s desire for ‘screen time’ on their participation

|  | **Negative Influence** | **Did Not Influence** | **Positive Influence** | Not Applicable |
| --- | --- | --- | --- | --- |

|  | 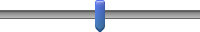 |
| --- | --- |

|  |
| --- |

The cost of the activity

|  | **Negative Influence** | **Did Not Influence** | **Positive Influence** | Not Applicable |
| --- | --- | --- | --- | --- |

|  |  |
| --- | --- |

End of Block: L2 Environmental

Start of Block: L2 Program

To what extent do you feel each of the following factors influenced participation in ${L2 A/ChoiceTextEntryValue/1}:

|  |
| --- |

This activity’s accommodation of my child’s behaviour

|  | **Negative Influence** | **Did Not Influence** | **Positive Influence** | Not Applicable |
| --- | --- | --- | --- | --- |

|  |  |
| --- | --- |

|  |
| --- |

How the activity makes my child feel

|  | **Negative Influence** | **Did Not Influence** | **Positive Influence** | Not Applicable |
| --- | --- | --- | --- | --- |

|  |  |
| --- | --- |

|  |
| --- |

Opportunities to start at beginner or introductory levels

|  | **Negative Influence** | **Did Not Influence** | **Positive Influence** | Not Applicable |
| --- | --- | --- | --- | --- |

|  |  |
| --- | --- |

|  |
| --- |

The flexibility of the activity (e.g. relaxed rules)

|  | **Negative Influence** | **Did Not Influence** | **Positive Influence** | Not Applicable |
| --- | --- | --- | --- | --- |

|  |  |
| --- | --- |

|  |
| --- |

The opportunity for social connection with peers, friends, parents or a buddy

|  | **Negative Influence** | **Did Not Influence** | **Positive Influence** | Not Applicable |
| --- | --- | --- | --- | --- |

|  |  |
| --- | --- |

|  |
| --- |

The use of flexible and alternative communication styles

|  | **Negative Influence** | **Did Not Influence** | **Positive Influence** | Not Applicable |
| --- | --- | --- | --- | --- |

|  |  |
| --- | --- |

|  |
| --- |

My partner, co-parent or spouse’s support of my child’s involvement in the activity

|  | **Negative Influence** | **Did Not Influence** | **Positive Influence** | Not Applicable |
| --- | --- | --- | --- | --- |

|  |  |
| --- | --- |

End of Block: L2 Program

Start of Block: L3: Considered

In the past five years what organised extracurricular social activities have **you considered involving your child in but never done so**

***We will ask you some follow up questions about the first activity listed***

*If no activity is relevant there is an option at the bottom of this page*

- Please list the name of one activity you considered   __________________________________________________

OR

- I have never considered involving my child in an organised extracurricular social activity

Add another organised extracurricular social activity **you have considered involving your child in but never done so**

- Yes
- No

End of Block: L3: Considered

Start of Block: L3 Behaviour

To what extent do you feel each of the following factors influenced participation in ${L3 A/ChoiceTextEntryValue/1}:

|  |
| --- |

My child’s communication skills

|  | **Negative Influence** | **Did Not Influence** | **Positive Influence** | Not Applicable |
| --- | --- | --- | --- | --- |

|  |  |
| --- | --- |

|  |
| --- |

My child’s motor skills

|  | **Negative Influence** | **Did Not Influence** | **Positive Influence** | Not Applicable |
| --- | --- | --- | --- | --- |

|  |  |
| --- | --- |

|  |
| --- |

My child’s social skills

|  | **Negative Influence** | **Did Not Influence** | **Positive Influence** | Not Applicable |
| --- | --- | --- | --- | --- |

|  |  |
| --- | --- |

|  |
| --- |

My child’s coordination

|  | **Negative Influence** | **Did Not Influence** | **Positive Influence** | Not Applicable |
| --- | --- | --- | --- | --- |

|  |  |
| --- | --- |

|  |
| --- |

My child’s attention while participating

|  | **Negative Influence** | **Did Not Influence** | **Positive Influence** | Not Applicable |
| --- | --- | --- | --- | --- |

|  |  |
| --- | --- |

|  |
| --- |

My child’s interests

|  | **Negative Influence** | **Did Not Influence** | **Positive Influence** | Not Applicable |
| --- | --- | --- | --- | --- |

|  |  |
| --- | --- |

|  |
| --- |

My child’s behaviour

|  | **Negative Influence** | **Did Not Influence** | **Positive Influence** | Not Applicable |
| --- | --- | --- | --- | --- |

|  |  |
| --- | --- |

|  |
| --- |

My child’s sensory preferences

|  | **Negative Influence** | **Did Not Influence** | **Positive Influence** | Not Applicable |
| --- | --- | --- | --- | --- |

|  |  |
| --- | --- |

End of Block: L3 Behaviour

Start of Block: L3 Social

To what extent do you feel each of the following factors influenced participation in ${L3 A/ChoiceTextEntryValue/1}:

|  |
| --- |

Attitudes of other parents towards me

|  | **Negative Influence** | **Did Not Influence** | **Positive Influence** | Not Applicable |
| --- | --- | --- | --- | --- |

|  |  |
| --- | --- |

|  |
| --- |

Attitudes of facilitators or coaches towards me

|  | **Negative Influence** | **Did Not Influence** | **Positive Influence** | Not Applicable |
| --- | --- | --- | --- | --- |

|  |  |
| --- | --- |

|  |
| --- |

Attitudes of other parents towards my child

|  | **Negative Influence** | **Did Not Influence** | **Positive Influence** | Not Applicable |
| --- | --- | --- | --- | --- |

|  |  |
| --- | --- |

|  |
| --- |

Attitudes of facilitators or coaches towards my child

|  | **Negative Influence** | **Did Not Influence** | **Positive Influence** | Not Applicable |
| --- | --- | --- | --- | --- |

|  |  |
| --- | --- |

|  |
| --- |

The skill level of facilitators or coaches

|  | **Negative Influence** | **Did Not Influence** | **Positive Influence** | Not Applicable |
| --- | --- | --- | --- | --- |

|  |  |
| --- | --- |

|  |
| --- |

The individual or group nature of the activity

|  | **Negative Influence** | **Did Not Influence** | **Positive Influence** | Not Applicable |
| --- | --- | --- | --- | --- |

|  |  |
| --- | --- |

|  |
| --- |

My child’s possible exposure to bullying

|  | **Negative Influence** | **Did Not Influence** | **Positive Influence** | Not Applicable |
| --- | --- | --- | --- | --- |

|  |  |
| --- | --- |

|  |
| --- |

My beliefs about the value of the activity for my child

|  | **Negative Influence** | **Did Not Influence** | **Positive Influence** | Not Applicable |
| --- | --- | --- | --- | --- |

|  |  |
| --- | --- |

End of Block: L3 Social

Start of Block: L3 Environmental

To what extent do you feel each of the following factors influenced participation in ${L3 A/ChoiceTextEntryValue/1}:

|  |
| --- |

The availability of appropriate and safe equipment and facilities

|  | **Negative Influence** | **Did Not Influence** | **Positive Influence** | Not Applicable |
| --- | --- | --- | --- | --- |

|  |  |
| --- | --- |

|  |
| --- |

The availability of transport

|  | **Negative Influence** | **Did Not Influence** | **Positive Influence** | Not Applicable |
| --- | --- | --- | --- | --- |

|  |  |
| --- | --- |

|  |
| --- |

My child’s familiarity with surrounds

|  | **Negative Influence** | **Did Not Influence** | **Positive Influence** | Not Applicable |
| --- | --- | --- | --- | --- |

|  |  |
| --- | --- |

|  |
| --- |

Our family’s availability

|  | **Negative Influence** | **Did Not Influence** | **Positive Influence** | Not Applicable |
| --- | --- | --- | --- | --- |

|  |  |
| --- | --- |

|  |
| --- |

The impact of my child’s desire for ‘screen time’ on their participation

|  | **Negative Influence** | **Did Not Influence** | **Positive Influence** | Not Applicable |
| --- | --- | --- | --- | --- |

|  |  |
| --- | --- |

|  |
| --- |

The cost of the activity

|  | **Negative Influence** | **Did Not Influence** | **Positive Influence** | Not Applicable |
| --- | --- | --- | --- | --- |

|  |  |
| --- | --- |

End of Block: L3 Environmental

Start of Block: L3 Program

To what extent do you feel each of the following factors influenced participation in ${L3 A/ChoiceTextEntryValue/1}:

|  |
| --- |

This activity’s accommodation of my child’s behaviour

|  | **Negative Influence** | **Did Not Influence** | **Positive Influence** | Not Applicable |
| --- | --- | --- | --- | --- |

|  |  |
| --- | --- |

|  |
| --- |

How the activity makes my child feel

|  | **Negative Influence** | **Did Not Influence** | **Positive Influence** | Not Applicable |
| --- | --- | --- | --- | --- |

|  |  |
| --- | --- |

|  |
| --- |

Opportunities to start at beginner or introductory levels

|  | **Negative Influence** | **Did Not Influence** | **Positive Influence** | Not Applicable |
| --- | --- | --- | --- | --- |

|  |  |
| --- | --- |

|  |
| --- |

The flexibility of the activity (e.g. relaxed rules)

|  | **Negative Influence** | **Did Not Influence** | **Positive Influence** | Not Applicable |
| --- | --- | --- | --- | --- |

|  |  |
| --- | --- |

|  |
| --- |

The opportunity for social connection with peers, friends, parents or a buddy

|  | **Negative Influence** | **Did Not Influence** | **Positive Influence** | Not Applicable |
| --- | --- | --- | --- | --- |

|  |  |
| --- | --- |

|  |
| --- |

The use of flexible and alternative communication styles

|  | **Negative Influence** | **Did Not Influence** | **Positive Influence** | Not Applicable |
| --- | --- | --- | --- | --- |

|  |  |
| --- | --- |

|  |
| --- |

My partner, co-parent or spouse’s support of my child’s involvement in the activity

|  | **Negative Influence** | **Did Not Influence** | **Positive Influence** | Not Applicable |
| --- | --- | --- | --- | --- |

|  |  |
| --- | --- |

End of Block: L3 Program

***Insert Debrief***
